# Neonatal Jaundice Requiring Phototherapy Risk Factors in a Newborn Nursery: Machine Learning Approach

**DOI:** 10.3390/children12081020

**Published:** 2025-08-01

**Authors:** Yunjin Choi, Sunyoung Park, Hyungbok Lee

**Affiliations:** Nursing Department, Seoul National University Hospital, Seoul 03038, Republic of Korea; preemielover@gmail.com (Y.C.); 17735@snuh.org (S.P.)

**Keywords:** neonatal jaundice, hyperbilirubinemia, phototherapy, machine learning, electronic medical records

## Abstract

**Highlights:**

**What are the main findings?**

- Machine learning algorithms successfully identified the key perinatal factors, including mode of delivery, feeding patterns, maternal BMI, and neonatal birth weight, that are associated with the risk of neonatal jaundice requiring phototherapy.
- Specifically, Cesarean section delivery, increased breastfeeding and formula intake, and lower birth weight were found to significantly increase the likelihood of neonates needing phototherapy for jaundice.

**What is the implication of the main finding?**

- The development of predictive models leveraging electronic medical records offers a powerful tool for early risk stratification, enabling timely clinical interventions and the more effective management of neonatal jaundice.
- These findings emphasize the critical need for integrating comprehensive maternal and neonatal health data into real-time decision-making tools to help reduce complications and readmissions related to hyperbilirubinemia.

**Abstract:**

**Background:** Neonatal jaundice is common and can cause severe hyperbilirubinemia if untreated. The early identification of at-risk newborns is challenging despite the existing guidelines. **Objective:** This study aimed to identify the key maternal and neonatal risk factors for jaundice requiring phototherapy using machine learning. **Methods:** In this study hospital, phototherapy was administered following the American Academy of Pediatrics (AAP) guidelines when a neonate’s transcutaneous bilirubin level was in the high-risk zone. To identify the risk factors for phototherapy, we retrospectively analyzed the electronic medical records of 8242 neonates admitted between 2017 and 2022. Predictive models were trained using maternal and neonatal data. XGBoost showed the best performance (AUROC = 0.911). SHAP values interpreted the model. **Results:** Mode of delivery, neonatal feeding indicators (including daily formula intake and breastfeeding frequency), maternal BMI, and maternal white blood cell count were strong predictors. Cesarean delivery and lower birth weight were linked to treatment need. **Conclusions:** Machine learning models using perinatal data accurately predict the risk of neonatal jaundice requiring phototherapy, potentially aiding early clinical decisions and improving outcomes.

## 1. Introduction

Neonatal jaundice is a condition characterized by yellowish discoloration of the skin and sclera in newborns due to elevated bilirubin levels. It occurs in approximately 60% of term neonates and 80% of preterm neonates within the first week of life, typically resolving spontaneously within 2 to 3 weeks [1,2,3]. However, in some cases, neonatal jaundice can progress to severe hyperbilirubinemia, which may lead to irreversible neurological damage [4]. Therefore, the early diagnosis of jaundice is crucial for preventing severe complications by maintaining bilirubin levels within a safe range and enabling timely interventions, such as phototherapy or treatment of underlying conditions [1,4,5,6].

Neonatal jaundice is among the leading causes of hospital readmission during the neonatal period [2]. In response, the American Academy of Pediatrics (AAP) in 2022 recommended that all neonates born at ≥35 weeks’ gestation undergo bilirubin screening and a clinical risk assessment for severe hyperbilirubinemia prior to discharge. Furthermore, parental education is crucial for empowering caregivers to monitor jaundice at home and seek timely medical care if symptoms progress [7]. Beyond visual inspection, identifying high-risk neonates is essential for the early detection and prevention of severe hyperbilirubinemia.

Studies have identified several key risk factors, including preterm birth, exclusive breastfeeding in the early neonatal period, glucose-6-phosphate dehydrogenase (G6PD) deficiency, ABO incompatibility, maternal alloimmunization, maternal obesity, conception via in vitro fertilization and embryo transfer (IVF-ET), delayed cord clamping, and a gestational age of 35–36 weeks. Furthermore, early-term neonates (37 to less than 39 weeks) have a higher likelihood of requiring phototherapy compared to full-term neonates (39 to less than 41 weeks) [8,9,10,11,12,13].

Although several studies have investigated the risk factors associated with neonatal jaundice [8,9,10,11,12,13], few have utilized machine learning techniques on large-scale, single-center datasets to identify the risk factors associated with jaundice requiring treatment. Therefore, this study aims to apply machine learning algorithms to analyze the key risk factors for neonatal jaundice, ultimately contributing to improved early diagnosis and preventive strategies.

## 2. Methods

This study is a retrospective study applying machine learning techniques to analyze the factors influencing neonatal jaundice in a single tertiary hospital. This study population includes neonates admitted to the well-baby nursery at a single tertiary hospital from 1 January 2017 to 31 December 2022 based on electronic medical records. Neonates admitted to the neonatal intensive care unit (NICU) after birth were excluded. To identify the risk factors of the neonatal jaundice requiring treatment, various maternal and neonatal factors were analyzed. Maternal factors included age, weight, BMI, white blood cell count, hemoglobin, platelet count, gestational diabetes, hypertension during pregnancy, maternal conditions such as hypothyroidism, and the use of oxytocin during labor. Neonatal factors included gestational age, prematurity, premature rupture of membrane, prolonged rupture of membrane, low birth weight, mode of delivery, Apgar score, meconium pass during delivery, cord neck around, umbilical cord length, delayed cord clamping, urination and defecation at birth, and birth weight. Additionally, neonatal factors such as head circumference, chest circumference, abdominal circumference, weight loss rate of the birth weight, daily formula intake, daily breast milk feeding frequency, daily urination frequency, and daily defecation frequency were extracted. The outcome variable for identifying neonatal jaundice requiring treatment was extracted from nursing records. In the study hospital, when a neonate’s transcutaneous bilirubin level was in the high-risk zone, phototherapy was administered following the AAP guidelines, and at that time, nurses recorded “phototherapy initiated” in the nursing records. Therefore, neonates with a nursing record indicating the initiation of phototherapy from birth until discharge were identified. Repeated nursing records for the same neonate were extracted based on the first record.

**Data Preparation:** Data extraction was performed from the Clinical Data Warehouse of Seoul National University Hospital, using de-identified data to prevent patient identification. To ensure security, data extraction and analysis were conducted using internal servers and an internal analysis cloud. Since the data consisted of mandatory input fields, there were no missing values. However, outliers caused by input errors in weight, height, and vital signs were replaced with the mean values. Data imbalance is typically addressed using two techniques: under-sampling, which reduces the majority class data, and over-sampling, which increases the minority class data. Under-sampling may result in the loss of valuable data, while over-sampling can lead to overfitting. To mitigate these drawbacks, the SMOTE-Tomek method, which combines both under-sampling and over-sampling, has recently been utilized. In this study, due to severe data imbalance, the SMOTE-Tomek technique was applied to prevent overfitting and prediction bias.

**Machine Learning:** For model development, the data were split into training and testing sets in an 8:2 ratio, and model validation was performed using 5-fold cross-validation. Logistic Regression, Support Vector Machine, Random Forest, and XGBoost algorithms were applied and compared. To evaluate the predictive performance and accuracy of the machine learning models, metrics such as accuracy, precision, recall, F1-measure, and the area under the ROC curve were used. The ROC curve area was compared to select the most optimal algorithm. Finally, SHAP values were used to identify the influencing factors and explain the prediction results of the selected algorithm (to explain the prediction result of the selected algorithm, SHAP values were used to identify the influencing factors).

**Ethical Considerations:** Prior to initiating this study, approval was obtained from the Institutional Review Board (IRB) of Seoul National University Hospital (H-2305-118-1434). Data were extracted from the Clinical Data Warehouse using electronic medical records. Additionally, personal information was anonymized, and any identifiable patient data were de-identified.

## 3. Results

A total of 8242 neonates were included in this study, with 1699 (20.6%) requiring phototherapy for neonatal jaundice. Table 1 presents the general characteristics of the study population, comparing the phototherapy group (*n* = 1699) with the non-phototherapy group (*n* = 6543). There was no significant difference in neonatal jaundice prevalence based on gender (*p* = 0.643). However, multiple pregnancies were associated with a lower phototherapy rate (19.5% vs. 21.5%, *p* = 0.031). Neonates who experienced weight loss exceeding 5% of birth weight had a significantly higher phototherapy rate (27.7% vs. 18.7%, *p* < 0.001). Maternal factors also influenced the phototherapy rates. Infants born to mothers with blood type O had a higher phototherapy rate than those with non-O blood types (22.5% vs. 19.9%, *p* = 0.012). Gestational hypertensive disorders (27.7% vs. 19.8%, *p* < 0.001), prior artificial miscarriage (25.6% vs. 20.2%, *p* = 0.002), and cesarean section delivery (36.5% vs. 7.0%, *p* < 0.001) were significantly associated with increased phototherapy requirements. Regarding neonatal factors, preterm birth (24.0% vs. 19.1%, *p* < 0.001), small-for-gestational-age status (23.9% vs. 19.3%, *p* < 0.001), and lower birth weight (mean ± SD: 2.79 ± 0.54 kg vs. 2.85 ± 0.50 kg, *p* < 0.001) were significantly associated with a higher phototherapy rate. Additionally, neonates in the phototherapy group had a greater number of defecations (6.18 ± 4.97 vs. 5.41 ± 3.27 per day, *p* < 0.001) and urinations (7.11 ± 1.53 vs. 5.90 ± 1.87 per day, *p* < 0.001) compared to the non-phototherapy group.

**Figure 1:**
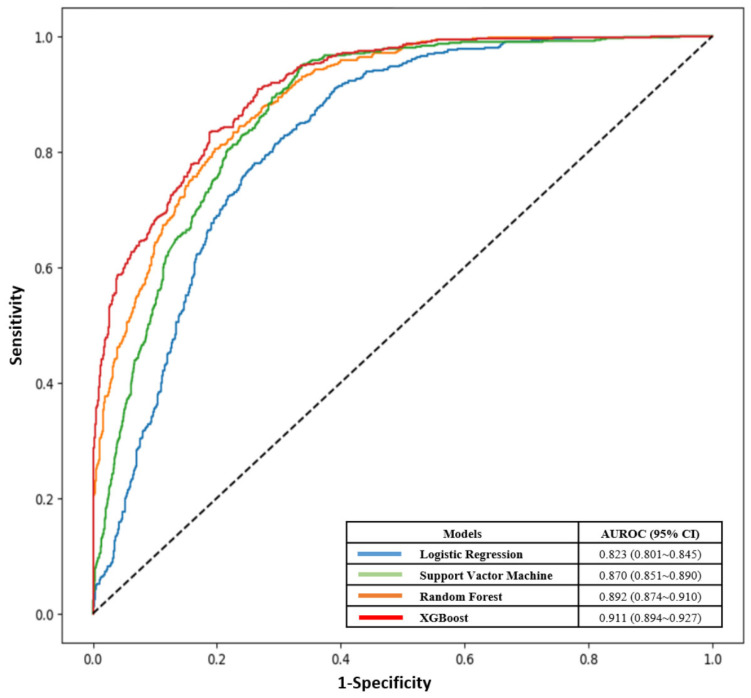
ROC curve of the 4 models.

The evaluation results of the machine learning models are shown in Table 2. The Logistic Regression had an accuracy of 0.754, precision of 0.632, recall of 0.566, F-1 measure of 0.597, and AUROC of 0.823 (95% CI: 0.801–0.845). The Support Vector Machine had an accuracy of 0.790, precision of 0.665, recall of 0.699, F-1 measure of 0.682, and AUROC of 0.870 (95% CI: 0.851–0.890). The Random Forest model had an accuracy of 0.815, precision of 0.710, recall of 0.716, F-1 measure of 0.713, and AUROC of 0.892 (95% CI: 0.874–0.910). The XGBoost model showed an accuracy of 0.828, precision of 0.758, recall of 0.713, F-1 measure of 0.726, and AUROC of 0.911 (95% CI: 0.894–0.927). The ROC curves for each model are shown in Figure 1. A comparison of the model evaluation results confirmed that the XGBoost model demonstrated the best predictive performance.

**Figure 2:**
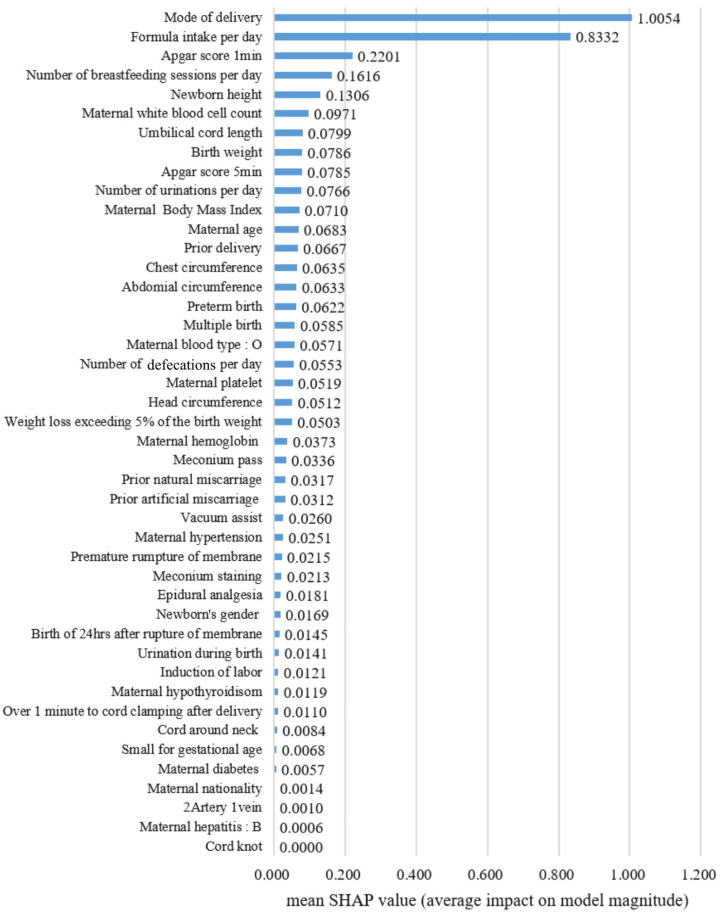
Feature importance.

The feature importance based on SHAP values for the XGBoost model, which demonstrated the best predictive performance, is shown in Figure 2. The factor that had the greatest impact on neonatal jaundice was the mode of delivery (mean SHAP value: 1.0054). This was followed by daily formula intake (mean SHAP value: 0.8332), the 1 min Apgar score (mean SHAP value: 0.2201), the daily breastfeeding sessions (mean SHAP value: 0.1616), neonatal height (mean SHAP value: 0.1306), and maternal white blood cell count (mean SHAP value: 0.0971).

**Figure 3:**
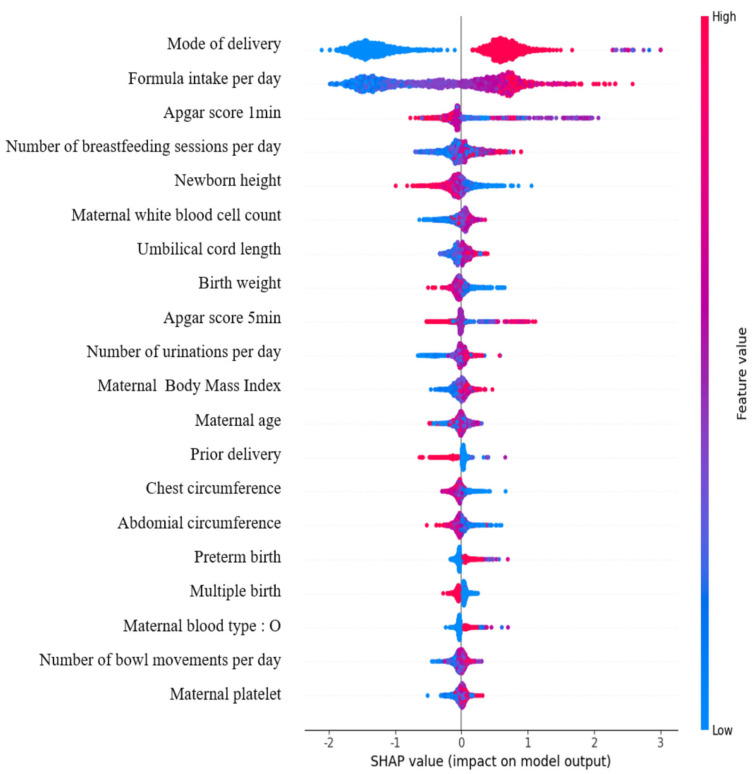
Top SHAP explainable model.

The SHAP explainable model for the top 20 factors influencing neonatal jaundice is shown in Figure 3. It was confirmed that neonates delivered via cesarean section were more likely to develop jaundice than those born through vaginal delivery. Additionally, higher daily formula intake and more frequent breastfeeding were associated with an increased likelihood of jaundice. Lower 1 min Apgar scores, lower neonatal birth weight, and shorter neonatal length were also linked to a higher occurrence of jaundice. Furthermore, higher maternal white blood cell counts, higher BMI, and older maternal age were associated with an increased likelihood of neonatal jaundice.

## 4. Discussion

This study applied machine learning techniques to analyze various factors influencing neonatal jaundice requiring phototherapy. This study revealed a higher incidence of jaundice in neonates delivered by cesarean section compared to vaginally delivered infants. This result stands in contrast to previous studies [14,15] that reported no statistically significant association between the type of delivery and the incidence of neonatal jaundice. While bilirubin levels generally reach their peak between 72 and 96 h postpartum, a common practice in numerous hospital settings involves discharging vaginally delivered newborns within 48 to 72 h, whereas neonates born via cesarean section typically remain hospitalized for a duration of 4 to 5 days. This divergence in hospitalization duration suggests that jaundice in cesarean-delivered infants is more likely to be detected during their hospital stay, whereas jaundice in vaginally delivered neonates is likely to be detected after they have been discharged. Thus, delayed detection and treatment of post-discharge jaundice may result in severe neurodevelopmental and long-term health complications [16]. Notably, numerous studies have established that neonatal hyperbilirubinemia significantly impacts neurodevelopment, irrespective of whether the infant is preterm or full-term [17]. Previous research has indicated that early discharge correlates with increased readmission rates for severe jaundice [18,19]. Furthermore, infants discharged following vaginal delivery are reportedly at greater risk of readmission due to hyperbilirubinemia [19]. Although clinical guidelines and parental education on jaundice detection after discharge are widely implemented, many neonates continue to experience complications from undetected jaundice. This may be attributed to the fact that the immediate postpartum period is a highly vulnerable time for mothers, characterized by psychological, physical, and cognitive challenges [20,21]. Therefore, the effectiveness of education provided during the initial postpartum hospitalization may be limited by maternal factors affecting retention and adherence [22,23]. Additionally, the lack of user-friendly and objective devices for parents to monitor their newborns’ bilirubin levels at home presents a significant challenge [24]. Recent studies have highlighted the potential of digital health interventions, such as smartphone-based bilirubin measurement, for frequent and noninvasive monitoring [24]. Nevertheless, the widespread commercial application of such technologies faces several limitations. Accordingly, to enable the early identification of neonatal jaundice post-discharge, a sustained effort in parental education from the antenatal stage, coupled with the development and validation of simple yet precise tools for at-home jaundice detection, is essential.

In this study, we found that nutrition-related variables during hospitalization, such as frequency of breastfeeding, daily formula intake, and frequency of urination, were associated with neonatal jaundice. Our observation that jaundice was more prevalent in newborns with a higher breastfeeding frequency aligns with the findings from prior research, supporting studies suggesting that frequent breastfeeding may increase the serum bilirubin levels [25,26]. Interestingly, in the present study, we also observed a positive correlation between increased formula intake, urination frequency, and the occurrence of neonatal jaundice. This observation could be explained by the fact that neonates with jaundice received intensified nutritional support through nursing interventions targeting bilirubin reduction [27], as per institutional protocols and clinical guidelines. Consequently, the interpretation of these nutrition-related variables should be approached with caution. While the World Health Organization (WHO) recommends exclusive breastfeeding for the first six months [28], the American Academy of Pediatrics (AAP) guidelines recognize the potential risk of breastfeeding-associated jaundice and advise careful monitoring of nutritional status [29]. Regional and institutional variations in practice [30,31] further underscore the necessity of standardized feeding guidelines. Therefore, further studies are needed to elucidate the intricate relationship between early feeding behaviors and the development of jaundice. Given the retrospective design of this study, prospective validation of the identified key nutritional variables is recommended.

Among maternal variables, both elevated maternal body mass index (BMI) and increased white blood cell (WBC) count prior to delivery were significantly associated with neonatal jaundice requiring treatment. These findings suggest that maternal metabolic and immunological status may influence neonatal bilirubin metabolism, consistent with previous studies reporting similar associations [8,32].

Additionally, neonatal factors such as lower birth weight, shorter birth length, and preterm birth were linked to an increased risk of jaundice. These results align with prior research indicating that immature hepatic function and underdeveloped physiology in preterm or growth-restricted neonates contribute to impaired bilirubin clearance [10].

Overall, our findings underscore the critical role of maternal health during pregnancy and the developmental maturity of the neonate in the effective metabolism of bilirubin. While previous studies have typically examined maternal and neonatal factors independently, this study employed a machine learning-based analysis of electronic medical record (EMR) data to integrate both.

Recently, machine learning-based software for neonatal jaundice prediction has been developed [33]. However, these models primarily utilize neonatal variables and currently focus on validating accuracy, clinical utility, and comparison with existing methods. Most existing machine learning approaches for predicting neonatal jaundice have focused on image-based analyses; however, integrated models utilizing EMR data remain limited [34,35,36,37]. This study highlights the potential of predictive modeling using routinely collected perinatal data—information that is readily available at the time of birth.

This study has several limitations. The retrospective design limits the establishment of causal relationships, necessitating prospective research. The use of single-center data may restrict the generalizability of the findings, suggesting the need for multi-center studies with diverse populations. Finally, this study did not account for post-discharge jaundice progression or complications such as neurodevelopmental disorders in neonates. Future research should include data on readmissions and complications for jaundice treatment to refine predictive models.

**Implications and Future Directions:** Our findings suggest that neonatal jaundice is a predictable condition that can be modeled using perinatal variables from both the mother and infant. These predictors—available at the time of delivery—could be leveraged to develop real-time decision support systems for jaundice risk stratification. Future research should focus on developing and validating large-scale artificial intelligence (AI) models incorporating diverse and comprehensive datasets. In particular, integrating post-discharge outcomes and feeding data may enhance model accuracy and real-world applicability. Such tools would be instrumental in reducing the clinical burden of neonatal jaundice and preventing avoidable complications through early intervention.

## 5. Conclusions

This study highlights the potential for developing a neonatal jaundice risk prediction model using machine learning. It is expected to serve as a valuable foundation for early identification and prevention strategies for neonatal jaundice. Moreover, we anticipate the development of strategies that not only prevent readmissions but also empower parents to identify and manage neonatal jaundice after discharge, thereby mitigating a range of potential complications. Further research can refine these findings and contribute to the development of a more sophisticated predictive model, ultimately helping to reduce the clinical burden of neonatal jaundice.

## Figures and Tables

**Table 1:** General characteristics.

**Characteristics**		**Non-Phototherapy Group** **(N = 6543)**		**Phototherapy Group** **(N = 1699)**		**x^2^**	***p***
Gender	M	3181	79.2%	837	20.8%	0.226	0.643
	F	3362	79.6%	862	20.4%		
Multiple pregnancies	No	3547	78.5%	971	21.5%	4.709	0.031
	Yes	2996	80.5%	728	19.5%		
Weight loss exceeding 5% of birth weight	No	5255	81.3%	1205	18.7%	70.188	<0.001
	Yes	1288	72.3%	494	27.7%		
Maternal country	Korea	6363	79.5%	1641	20.5%	2.113	0.144
	Other	180	75.6%	58	24.4%		
Maternal ABO blood group	Non-O	4819	80.1%	1199	19.9%	6.495	0.012
	O	1724	77.5%	500	22.5%		
Maternal HBsAg positive	No	6437	79.3%	1677	20.7%	0.933	0.379
	Yes	106	82.8%	22	17.2%		
Gestational DM	No	5978	79.6%	1529	20.4%	3.120	0.085
	Yes	565	76.9%	170	23.1%		
Gestational hypertensive disorders	No	5932	80.2%	1465	19.8%	28.827	0.000
	Yes	611	72.3%	234	27.7%		
Maternal thyroid disease	No	6113	79.6%	1566	20.4%	3.344	0.075
	Yes	430	76.4%	133	23.6%		
Premature rupture of membrane	No	5596	78.6%	1521	21.4%	18.279	<0.001
	Yes	947	84.2%	178	15.8%		
Parity	1	4782	78.5%	1310	21.5%	11.296	0.001
	2+	1761	81.9%	389	18.1%		
Prior artificial miscarriage	0	6111	79.8%	1550	20.2%	9.670	0.002
	1+	432	74.4%	149	25.6%		
Prior natural miscarriage	0	4921	79.4%	1275	20.6%	0.020	0.900
	1+	1622	79.3%	424	20.7%		
Induction of labor	No	3165	71.2%	1281	28.8%	396.5	<0.001
	Yes	3378	89.0%	418	11.0%		
Epidural analgesia	No	5564	77.8%	1592	22.2%	88.514	<0.001
	Yes	979	90.1%	107	9.9%		
Delayed cord clamping	No	6380	79.2%	1676	20.8%	7.911	0.004
	Yes	163	87.6%	23	12.4%		
Type of delivery	Normal	4125	93.0%	309	7.0%	1091.884	<0.001
	Cesarean section	2418	63.5%	1390	36.5%		
Vacuum assist	No	5157	76.7%	1566	23.3%	160.01	<0.001
	Yes	1386	91.2%	133	8.8%		
Small for gestational age	No	4788	80.7%	1148	19.3%	21.053	<0.001
	Yes	1755	76.1%	551	23.9%		
Preterm birth	No	4565	80.9%	1076	19.1%	25.881	<0.001
	Yes	1978	76.0%	623	24.0%		
Meconium pass	No	5069	77.3%	1485	22.7%	81.699	<0.001
	Yes	1474	87.3%	214	12.7%		
Meconium staining	No	6128	79.0%	1633	21.0%	14.829	<0.001
	Yes	415	86.3%	66	13.7%		
Cord around neck	No	5262	78.7%	1427	21.3%	11.233	0.001
	Yes	1281	82.5%	272	17.5%		
Cord knot	No	6503	79.5%	1682	20.5%	2.976	0.064
	Yes	40	70.2%	17	29.8%		
Umbilical cord vessels	2 arteries 1 vein	6497	79.4%	1688	20.6%	0.061	0.480
	1 artery 1 vein	46	80.7%	11	19.3%		
Urination during birth	No	5237	79.8%	1324	20.2%	3.704	0.058
	Yes	1306	77.7%	375	22.3%		
Prolonged rupture of membrane ^1^	No	6316	79.3%	1645	20.7%	0.347	0.600
	Yes	227	80.8%	54	19.2%		
**Characteristics**	**Non-Phototherapy Group** **(N = 6543)**		**Phototherapy Group** **(N = 1699)**			**F**	***p***
	**Mean**	**SD**	**Mean**	**SD**			
Birth weight	2.85	±0.50	2.79	±0.54		3.915	<0.001
Birth height	48.01	±2.31	47.47	±2.39		8.537	<0.001
Head circumference	33.85	±5.39	33.70	±1.77		1.098	0.272
Chest circumference	30.57	±2.12	30.43	±2.32		2.281	0.023
Abdominal circumference	28.32	±4.08	28.16	±2.39		1.513	0.130
Number of defecations (per day)	5.41	±3.27	6.18	±4.97		−7.665	<0.001
Number of urinations (per day)	5.90	±1.87	7.11	±1.53		−27.541	<0.001
Number of breastfeeding sessions (per day)	2.57	±3.59	2.21	±3.09		3.766	<0.001
Formula intake (per day)	166.85	±55.79	218.46	±54.3		−34.153	<0.001
Weight loss rate of the birth weight	3.53	±1.87	4.00	±2.00		−9.153	<0.001
Maternal age	40.24	±4.27	40.51	±4.24		−2.302	0.021
Maternal body mass index	27.33	±6.63	28.00	±5.87		3.361	<0.001
Maternal white blood cell count	8.51	±12.23	8.47	±2.19		0.136	0.892
Maternal hemoglobin	11.94	±2.04	12.11	±4.75		−2.240	0.025
Maternal platelet count	207.97	±65.44	212.90	±61.27		−2.807	0.005
Apgar score 1 min	7.88	±0.86	7.77	±0.99		4.339	<0.001
Apgar score 5 min	9.03	±0.56	9.00	±0.59		2.061	0.039
Umbilical cord length	50.20	±33.01	49.11	±26.17		1.251	0.211

^1^ birth of over 24 h after rupture of membrane.

**Table 2:** Comparison of the models.

Models	Accuracy	Precision	Recall	F1-Score	AUROC (95% CI)
Logistic Regression	0.754	0.632	0.566	0.597	0.823 (0.801~0.845)
Support Vactor Machine	0.79	0.665	0.699	0.682	0.870 (0.851~0.890)
Random Forest	0.815	0.710	0.716	0.713	0.892 (0.874~0.910)
XGBoost	0.827	0.739	0.713	0.726	0.911 (0.894~0.927)

## Data Availability

This study used electronic health record data (de-identified) from the Seoul National University Hospital. The dataset used in this study is not publicly available due to its sensitive nature, and the data use agreement condition. However, aggregated analysis results are available upon request.

## References

[1] Ansong-Assoku B., Adnan M., Daley S.F., Ankola P.A. (2024). Neonatal jaundice. StatPearls [Internet].

[2] Jardine L.A., Woodgate P. (2012). Neonatal jaundice. Am. Fam. Physician.

[3] Mitra S., Rennie J. (2017). Neonatal jaundice: Aetiology, diagnosis, and treatment. Br. J. Hosp. Med..

[4] Khurshid F., Rao S.P., Sauve C., Gupta S. (2022). Universal screening for hyperbilirubinemia in term healthy newborns at discharge: A systematic review and meta-analysis. J. Glob. Health.

[5] National Institute for Health and Care Excellence (2010). Jaundice in Newborn Babies Under 28 Days: NICE Guidelines. CG98. https://www.nice.org.uk/guidance/cg98.

[6] Rennie J., Burman-Roy S., Murphy M.S. (2010). Neonatal jaundice: Summary of NICE guidance. BMJ.

[7] Kemper A.R., Newman T.B., Slaughter J.L., Maisels M.J., Watchko J.F., Downs S.M., Grout R.W., Bundy D.G., Stark A.R., Bogen D.L. (2022). Clinical Practice Guideline Revision: Management of Hyperbilirubinemia in the Newborn Infant 35 or More Weeks of Gestation. Pediatrics.

[8] Kaur N., Dhillon G., Sasidharan S., Dhillon H. (2021). Maternal and neonatal risk factors for neonatal jaundice and readmission—An Indian perspective. Acta Medica Int..

[9] Lin Q., Zhu D., Chen C., Feng Y., Shen F., Wu Z. (2022). Risk factors for neonatal hyperbilirubinemia: A systematic review and meta-analysis. Transl. Pediatr..

[10] Lee B.K., Le Ray I., Sun J.Y., Wikman A., Reilly M., Johansson S. (2016). Haemolytic and nonhaemolytic neonatal jaundice have different risk factor profiles. Acta Paediatr..

[11] Tan T.-J., Chen W.-J., Lin W.-C., Yang M.-C., Tsai C.-C., Yang Y.-N., Yang S.-N., Liu H.-K. (2023). Early-Term Neonates Demonstrate a Higher Likelihood of Requiring Phototherapy Compared to Those Born Full-Term. Children.

[12] Rehna T., Thomas S.A. (2022). Risk factors for early hyperbilirubinemia in neonates: A cross-sectional study. J. Curr. Res. Sci. Med..

[13] Qattea I., Farghaly M.A.A., Elgendy M., Mohamed M.A., Aly H. (2022). Neonatal hyperbilirubinemia and bilirubin neurotoxicity in hospitalized neonates: Analysis of the US database. Pediatr. Res..

[14] Garosi E., Mohammadi F., Ranjkesh F. (2016). The relationship between neonatal jaundice and maternal and neonatal factors. Iran. J. Neonatol..

[15] De Luca D., Carnielli V.P., Paolillo P. (2009). Neonatal hyperbilirubinemia and early discharge from the maternity ward. Eur. J. Pediatr..

[16] Maisels M.J. (2009). Neonatal hyperbilirubinemia and kernicterus—Not gone but sometimes forgotten. Early Hum. Dev..

[17] Merino-Andrés J., Pérez-Nombela S., Álvarez-Bueno C., Hidalgo-Robles Á., Ruiz-Becerro I., Fernández-Rego F.J. (2024). Neonatal hyperbilirubinemia and repercussions on neurodevelopment: A systematic review. Child Care Health Dev..

[18] Blumovich A., Mangel L., Yochpaz S., Mandel D., Marom R. (2020). Risk factors for readmission for phototherapy due to jaundice in healthy newborns: A retrospective, observational study. BMC Pediatr..

[19] Lain S.J., Roberts C.L., Bowen J.R., Nassar N. (2015). Early discharge of infants and risk of readmission for jaundice. Pediatrics.

[20] Mercer R.T. (2006). Nursing support of the process of becoming a mother. J. Obstet. Gynecol. Neonatal Nurs..

[21] Henry J.F., Sherwin B.B. (2012). Hormones and cognitive functioning during late pregnancy and postpartum: A longitudinal study. Behav. Neurosci..

[22] Dietz C., Swinkels S.H.N., van Daalen E., van Engeland H., Buitelaar J.K. (2007). Parental Compliance After Screening Social Development in Toddlers. Arch. Pediatr. Adolesc. Med..

[23] Kinshella M.-L.W., Salimu S., Chiwaya B., Chikoti F., Chirambo L., Mwaungulu E., Banda M., Hiwa T., Vidler M., Molyneux E.M. (2022). Challenges and recommendations to improve implementation of phototherapy among neonates in Malawian hospitals. BMC Pediatr..

[24] Hegde D., Rath C., Amarasekara S., Saraswati C., Patole S., Rao S. (2023). Performance of smartphone application to accurately quantify hyperbilirubinemia in neonates: A systematic review with meta-analysis. Eur. J. Pediatr..

[25] Soldi A., Tonetto P., Varalda A., Bertino E. (2011). Neonatal jaundice and human milk. J. Matern.-Fetal Neonatal Med..

[26] Gao C., Guo Y., Huang M., He J., Qiu X. (2023). Breast milk constituents and the development of breast milk jaundice in neonates: A systematic review. Nutrients.

[27] Ma X.W., Fan W.Q. (2020). Earlier nutrient fortification of breast milk fed low birth weight infants improves jaundice related outcomes. Nutrients.

[28] WHO (2009). Baby-Friendly Hospital Initiative: Revised, Updated and Expanded for Integrated Care (Section 3). Wellstart International. https://www.who.int/publications/i/item/9789241594950.

[29] American Academy of Pediatrics Subcommittee on Hyperbilirubinemia (2004). Management of hyperbilirubinemia in the newborn infant 35 or more weeks of gestation. Pediatrics.

[30] Flaherman V.J., Maisels M.J. (2017). ABM Clinical Protocol #22: Guidelines for management of jaundice in the breastfeeding infant 35 weeks or more of gestation—Revised 2017. Breastfeed. Med..

[31] Wilde V.K. (2021). Breastfeeding insufficiencies: Common and preventable harm to neonates. Curēus.

[32] Tavakolizadeh R., Izadi A., Seirafi G., Khedmat L., Mojtahedi S.Y. (2018). Maternal risk factors for neonatal jaundice: A hospital-based cross-sectional study in Tehran. Eur. J. Transl. Myol..

[33] Steffens B., Koch G., Engel C., Franz A.R., Pfister M., Wellmann S. (2025). Assessing accuracy of BiliPredics algorithm in predicting individual bilirubin progression in neonates—Results from a prospective multi-center study. Front. Digit. Health.

[34] Norman M., Aberg K., Holmsten K., Weibel V., Ekeus C. (2015). Predicting nonhemolytic neonatal hyperbilirubinemia. Pediatrics.

[35] Olusanya B.O., Osibanjo F.B., Slusher T.M. (2015). Risk factors for severe neonatal hyperbilirubinemia in low and middle-income countries: A systematic review and meta-analysis. PLoS ONE.

[36] Guedalia J., Farkash R., Wasserteil N., Kasirer Y., Rottenstreich M., Unger R., Grisaru Granovsky S. (2022). Primary risk stratification for neonatal jaundice among term neonates using machine learning algorithm. Early Hum. Dev..

[37] Daunhawer I., Kasser S., Koch G., Sieber L., Cakal H., Tütsch J., Pfister M., Wellmann S., Vogt J.E. (2019). Enhanced early prediction of clinically relevant neonatal hyperbilirubinemia with machine learning. Pediatr. Res..

